# *In-vitro* evaluation of the shear bond strength and fluoride release of a new bioactive dental composite material

**DOI:** 10.4317/jced.58966

**Published:** 2022-01-01

**Authors:** Hasna Rifai, Syed Qasim, Syed Mahdi, Martijn-Jacky Lambert, Ralph Zarazir, Fransesco Amenta, Sara Naim, Carina Mehanna

**Affiliations:** 1 Faculty of Dentistry,Saint Joseph University of Beirut, Beirut, Lebanon; 2 Faculty of Dentistry, University of Kuwait, Kuwait City 12037, Kuwait; 3 Center of Clinical Research, Telemedicine & Telepharmacy Department, School of Medicinal and Health Products Sciences, University of Camerino, 62032 Camerino, Italy; 4 Department of Community Dentistry, Jinnah Medical and Dental College, Sohail University, Karachi 74800, Pakistan; 5 Department of Copmmunity Dentistry and Oral Public Health, Special Needs in Oral Health, Dental School, Ghent University, Ghent, Belgium; 6 Department of Restorative and Esthetic Dentistry, Dentistry, Faculty of Dental medicine, Saint Joseph University of Beirut, Beirut, Lebanon; 7 Center of Clinical Research, Telemedicine & Telepharmacy Department, School of Medicinal and Health Products Sciences, University of Camerino, 62032 Camerino, Italy; 8 Postgraduate Program Department of Esthetic and Restorative Dentistry, School of Dentistry, Saint Joseph University of Beirut, Beirut, Lebanon

## Abstract

**Background:**

The aim of this study was (1) to determine and compare the shear bond strength (SBS) of a bioactive composite “Activa Bioactive Restorative” with and without bonding agent and a nanocomposite “Filtek Z350 XT/Z350” and (2) to measure and compare the amount of fluoride release from a bioactive composite “Activa Bioactive Restorative” and a glass ionomer ”Equia forte”.

**Material and Methods:**

Forty two dentin surfaces from freshly extracted human molars were prepared for shear bond strength testing. The specimens were randomly divided into three equal groups. The restorative materials were applied to all dentin surfaces according to the manufacturer’s instructions, using a special jig (Ultradent) in the following manner : Group 1 (Activa Bioactive Restorative with adhesive), Group 2 (Activa Bioactive Restorative without adhesive) and Group 3 (Filtek Z350 XT/Z350). The bonded specimens were subjected to thermocycling in 5°C and 55°C water baths then tested for SBS in a universal testing machine (1 mm/minute). Kolmogorov-Smirnov and Levene tests were used to evaluate the distribution of the variable and the equality of variances respectively and a Student’s T- test was applied to compare the mean strength between the groups. In the next test, thirty disc shaped specimens were fabricated using Activa BioActive restorative and Equia Forte; 15 specimens from each material. The specimens of each group were immersed separately in 5 ml of deionized water. Fluoride release was measured daily throughout 15 days using a fluoride-specific ion electrode and an ion-analyzer. Repeated measures analysis of variance with one within-subject factor (time) and one between-subject factor (Activa Bioactive / Equia Forte) was applied to compare the amount of released fluoride between groups and within time. It was followed by univariate analyses and Bonferroni multiple comparisons tests.

**Results:**

The mean shear bond strength of Activa Bioactive Restorative with adhesive was found to be 17.379 (± 8.5043) MPa and 19.443(± 8.3293) MPa for the Filtek Z350 XT/Z350 group. There was no significant difference between both groups. Regarding fluoride release, the amount of Fluoride released was significantly greater in the Equia Forte group compared to the Activa Bioactive group (-*p-value*<0.05). The mean amount of Fluoride has significantly decreased over time with Activa Bioactive group (-*p-value*<0.001); it showed the highest fluoride release during the first 24 hours post-setting. Also in the Equia Forte group, the mean amount of Fluoride release showed a progressive and significant decrease over time (-*p-value*<0.001), although the amount of Fluoride released was significantly greater in the Equia Forte group compared to the Activa Bioactive group (-*p-value*<0.05).

**Conclusions:**

Activa Bioactive Restorative with adhesive and a nanocomposite showed similar bond strengths. Activa Bioactive Restorative doesn’t have the self-adhesive property. The fluoride ion release profile of Activa was lower than that of the Equia Forte.

** Key words:**Bioactive composite, nanocomposite, glass ionomer, fluoride release, shear bond strength, thermocycling.

## Introduction

Dental caries is one of the major oral health problems met by dental professionals (1). In order to cope with tooth decay, today’s focus is shifting towards a more conservative strategy, saving tooth structure by using restorative materials which adhere to tooth tissues and require minimal interventions (2). In most clinical cases, caries removal results in large areas of exposed dentin (3). While bonding to enamel is reliable through micromechanical retention (4), dentinal bonding is still somewhat problematic, mainly because dentin is a “vital” tissue with high water and organic content (vs enamel) and it has a microstructure dominated by tubules (5). Therefore, satisfactory attributes should be demonstrated by the restorative materials at the tooth- material interface (6,7).

Glass ionomers (GIC) have been used for decades in restorative dentistry (8). They bond directly to teeth and have remineralizing properties on dental tissues through continuous fluoride release (9,10). The main disadvantages of using GIs as permanent restorations include 

their susceptibility to water uptake and loss, especially in the initial setting reaction, as well as their brittle material character (11,12). However, highly viscous GIs are always being improved in terms of clinical handling and performance. Consequently, some of them can be used in restorative dentistry as a permanent restorative material (13). Developments in the material sciences recently led to the launching of a glass hybrid product -“Equia Forte”- consisting of a highly viscous conventional GIC combined with a nanofilled coating material. It showed a quite satisfying clinical performance (14).

Lately, nanotechnology has been used in the composition of new types of resin composites (5). These have become the first choice direct restorative material in dentistry, due to the possibility to perform minimally invasive, or non-invasive treatments, associated with favorable properties and reliable clinical performance (15). They have numerous advantages such as: favorable physical and mechanical properties, including high resistance to compression and wear, relatively low costs and simple application (16). Despite the improvements in the dental composites’ technology, they still have limited lifetime. Dynamic changes in pH and temperature in the oral cavity due to diet, saliva and aging lead to degradation in the resin composite during clinical service (17,18). In addition, the biggest limitation or disadvantage of dental nanocomposites is that they have a polymerization shrinkage, possibly inducing marginal leakage, tooth or restoration fracture, or postoperative sensitivity, all leading to the reduction of the long-term success of the restoration (19). When composite restorations fail, they are mostly treated by restoration replacement, resulting in additional and progressive tissue loss throughout time (20).

To increase the restoration’s resistance to recurrent caries, agents can be added to affect remaining micro-organisms and/or to enhance remineralization of demineralized hard dental tissues (3). The battle against secondary caries, being as old as composite restorations themselves, and the quest of less invasive preparation techniques are the primary motivation behind the studies of bioactive composites (21). Ideal properties of a bioactive material with specific indications for dentistry include stimulating reparative dentin formation, bactericidal or bacteriostatic activity, and the maintenance of pulpal vitality (22). Bioactive materials are used as treatments for caries intervention, tooth structure remineralization, and bone regenerations (23). In addition, bioactive materials, compared with bulk-fill resin based composites, have minimal polymerization shrinkage (24).

A new type of bioactive resin- based composite (Activa Bioactive Restorative) was launched globally in 2013. It is described by the manufacturer as a bioactive self-adhesive material (25). It is a highly innovative, biologically agile and an esthetically pleasing composite material that incorporates all the advantages of GI cements in a strong, resilient resin matrix (26). This material constitutes of a blend of diurethane and methacrylate-based monomers with a modified polyacrylic acid and polybutadiene modified diurethane dimethacrylate (27). It contains no Bisphenol A, Bis-GMA or BPA derivatives (28). According to the manufacturer, the triple setting mode of this material includes the acid-base neutralization reaction of GICs plus the self-cure and the light-cure modes of the matrix (29).

Therefore, the aim of this *in vitro* study was to comparatively evaluate the shear bond strength between a new bioactive material ‘’Activa Bioactive Restorative” with and without adhesive and a nanocomposite ‘’Filtek Z350XT/Z350 ‘’; and to measure and compare the amount of fluoride release from this bioactive composite “Activa Bioactive Restorative” and a glass ionomer ”Equia forte”.

The first null hypothesis was that there is no difference in the mean shear bond strength between Activa Bioactive Restorative with and without adhesive.

The second null hypothesis was that there is no difference in the mean shear bond strength between Activa Bioactive Restorative with adhesive and Filtek Z350 XT/Z350.

The third null hypothesis was that there is no significant difference in fluoride release from Activa BioActive restorative and Equia Forte.

## Material and Methods

Dental materials used in the study, according to the data provided by the manufacturers, are specified in Table 1.

**Table 1:**
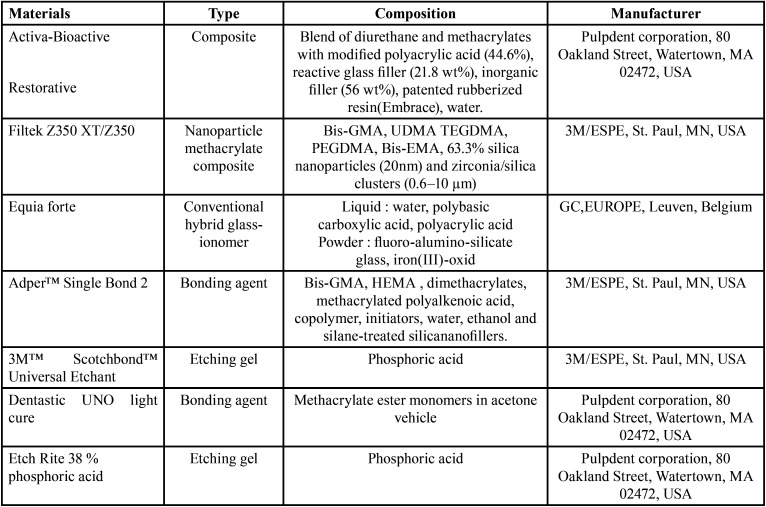
Dental materials used in the study.

-Shear Bond Strength

15 freshly extracted permanent molar teeth free of caries, cracks, attrition, abrasion, or restorations were collected. The teeth were cleaned of calculus and soft tissues and stored in distilled water till the study was conducted.

**Figure 1:**
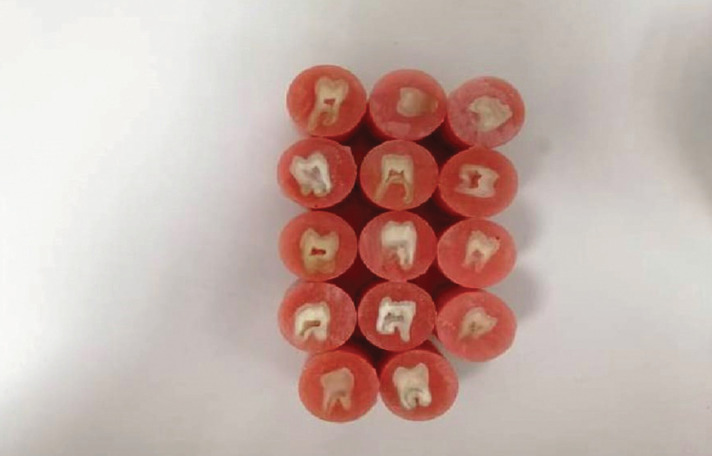
(a) preoperative view of the patient (frontal view); (b) claw-like position of patient’s hand; (c) maximal mouth opening; (d) mouth opening measuring 26mm.

The teeth were longitudinally sectioned in a mesiodistal direction (2 mm thickness) using a diamond disc with copious water spray. 42 sectioned surfaces with large dentinal surfaces were embedded in auto-polymerizing acrylic. After polymerization of the embedding resin, the surfaces were abraded then polished in a polishing machine (Fig. 1).

**Figure 2:**
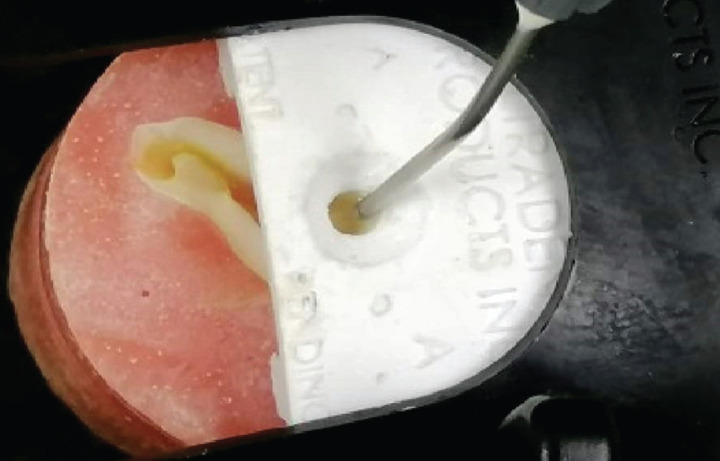
Panoramic radiography.

**Figure 3:**
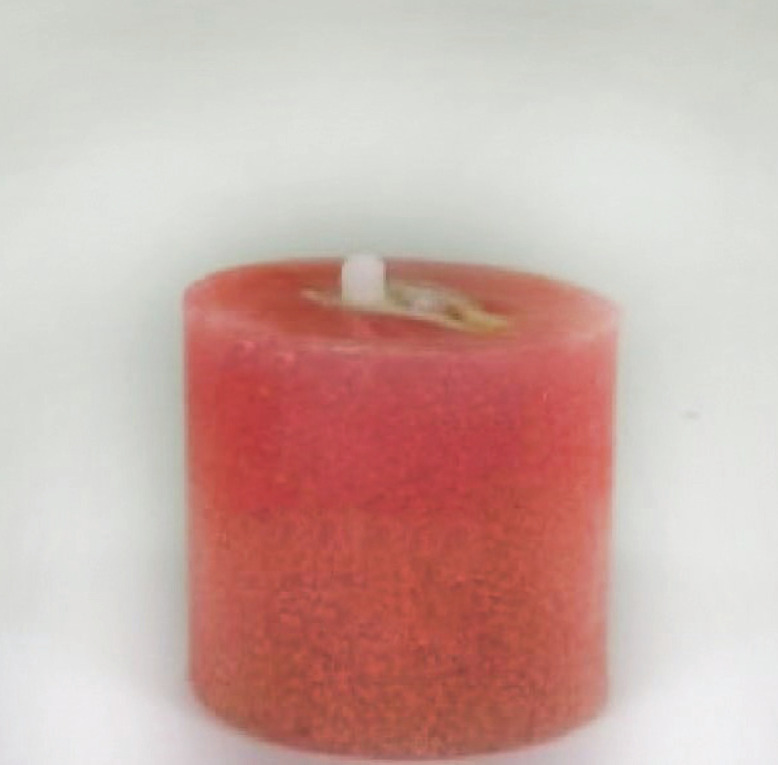
(a) modified upper tray; (b) maxillary preliminary impression; (c) final impression, (d) maxillary master cast.

All samples were stored in distilled water at room temperature and randomly divided into 3 groups of 14 teeth each. A restoration was placed on the dentin surface by filling a split Teflon mold with a cylindrical opening, tightly clamped to the embedded tooth, according to one of the following procedures : Activa BioActive Restorative with adhesive, Activa Bioactive Restorative without adhesive, Filtek Z350 XT/Z350 (Figs. 2,3).

All specimens were light-cured using a halogen light-curing unit at 1000 mw/cm2 (Dentastic UNO light (Pulpdent)).

• Group 1 (Activa BioActive with bonding)

Tooth surfaces were conditioned for 10 seconds using Etch-Rite 38% phosphoric acid etching gel (Pulpdent). The surfaces were rinsed for 20s with water and blot dried. Dentastic UNO light (Pulpdent) was applied and light cured for 10 seconds. Activa restorative material was inserted in the Teflon mold and then light- polymerized for 20 seconds.

• Group 2 (Activa BioActive without bonding)

Tooth surfaces were conditioned for 10 seconds using Etch-Rite 38% phosphoric acid etching gel (Pulpdent). The surfaces were rinsed for 20s with water and blot dried. Activa restorative was inserted in the Teflon mold then light polymerized for 20 seconds.

• Group 3 (Filtek Z350 XT/Z350) 

Tooth surfaces were conditioned using Scotchbond ™ Universal Etchant. The surfaces were rinsed with water and blot dried. Two consecutive coats of Adper™ Single Bond 2 were applied, followed by gentle air- drying and then light- polymerized for 10 seconds. Filtek Z350 XT/Z350 was placed in the Teflon mold and light cured for 20 seconds.

It is important to note that during the realization of group 2(Activa BioActive without bonding), the resins came off from all the teeth surfaces when removing the plastic plate and before exerting any force on these restorations, that’s why this group has been excluded from the experiment.

The bonded specimens (group1 & group 3) were stored in distilled water at room temperature then subjected to thermocycling in a water bath for 1000 cycles between 5 and 55°C with a dwell time of 30 seconds in each bath and transfer time of 10 seconds.

Shear bond testing was performed using a knife-edge blade in Ultratester (Ultradent products, Utah,USA) that was applied at the tooth-restoration interface at a crosshead speed of 1mm/min.

The SBS values were calculated in MegaPascals (MPa). The SBS was recorded and data obtained were subjected to statistical analysis.

The statistical analyses were performed using IBM SPSS Statistics (version 25.0). The level of significance was set at -*p-value* ≤0.05. Kolmogorov-Smirnov test revealed that the Shear Bond Strength was normally distributed among groups and Levene test revealed the equality of variances. Independent Student t tests was therefore used to compare continuous variables between the two groups.

-Fluoride release

**Figure 4:**
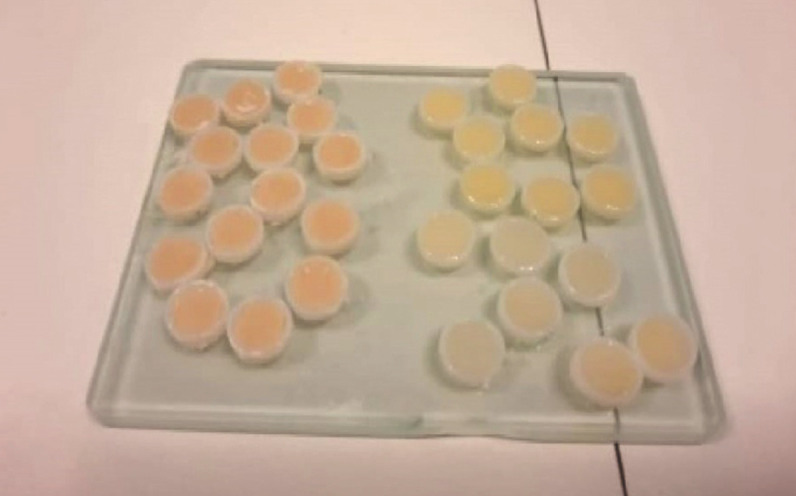
(a) wax rim representing patient’s occlusion, (b) denture reinforced with stainless steel mesh, (c) final denture, (d)removing denture in special manner.

Thirty specimens, in the shape of 8 mm diameter and 2 mm thick discs, were prepared of each of the two materials ( Equia Forte, Activa Bioactive Restorative), 15 per group (Fig. 4).

Molds were placed on a mylar strip and a glass slab, filled with Equia Forte or Activa Bioactive Restorative, covered with another glass slide to eliminate excess material were used. Activa Bioactive Restorative were light-cured for 20 s; Equia Forte were allowed to set for 2 min 30 s. After setting of the material, the specimens were freed from the molds then individually immersed in a vial containing 5 ml of deionized water and stored at a constant temperature of 37°C for 24h.

After 24 h, the samples were removed from the vials and the concentration of released fluoride ions were measured using a fluoride-specific ion electrode connected to a digital ion analyzer. Specimens were rinsed, dried, then immersed into a new vial containing 5 ml of deionized water. Between measurements, the electrode was rinsed with deionized water. Measurements were studied every 24 h for 15 consecutive days. Each vial was labelled, so the discs could be traced individually during the study period.

Fluoride release in the water was measured by its electric conduction in mV. As a standard comparison, 5 solutions with a fixed and well-known fluoride concentration of 160ppm, 48ppm, 32ppm, 1.6ppm, 0.48ppm were prepared and tested for their ion conduction in mV. In order to have a precise measurement, each of these standard solutions was tested 10 times, and the mean value was set as standard value. This value in mV enables us to mathematically convert the measurements in mV of the water vials with unknown fluoride concentration into its corresponding concentration in ppm.

The statistical analyses were performed then, using IBM SPSS Statistics (version 26.0). The level of significance was set at -*p-value* ≤0.05. Repeated measures analysis of variance with one within-subject factor (time) and one between-subject factor (Activa Bioactive / Equia Forte) was applied to compare the amount of released fluoride between groups and within time. It was followed by univariate analyses and Bonferroni multiple comparisons tests.

## Results

-Shear Bond Strength

**Figure 5:**
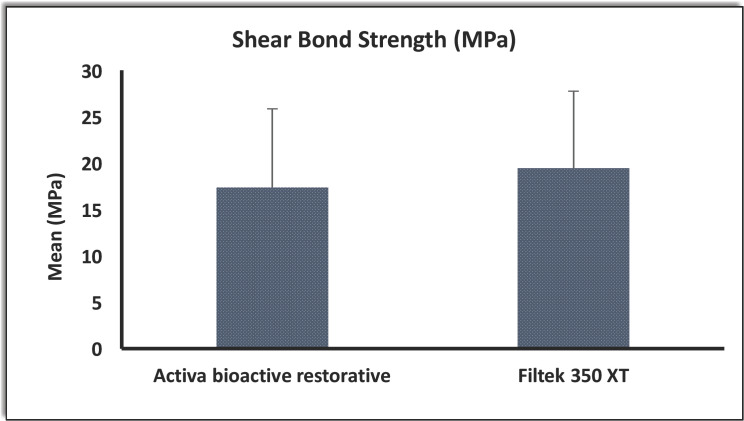
Mean of shear bond strength of the restorative materials.

Bond strength of Activa bioactive restorative ranged from 2.6 MPa to 33.0 MPa, with a mean of 17.379 ± 8.5043 MPa. The Filtek 350 XT group had bond strength values from 7.4 MPa to 38.0 MPa , with a mean of 19.443 ± 8.3293 MPa The bioactive restorative material had a lower mean bond strength than the Filtek 350 XT, but this difference was not statistically significant (-*p-value*=0.522) (Table 2, Fig. 5).

**Table 2:**
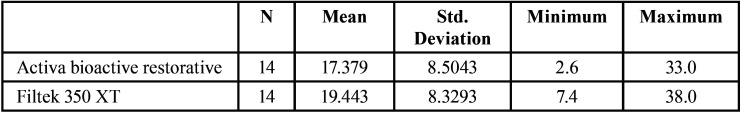
Mean Shear Bond Strength among groups.

-Fluoride release

The mean amount of Fluoride has significantly decreased over time with Activa Bioactive group (-*p-value* <0.001); it was 19.720 ± 1.317ppm on Day 1 and 0.032 ± 0.013 ppm on Day 15. The amount decreased considerably between Day 1 and Day 2 (-*p-value* <0.001), then between Day 2 and Day 3 (-*p-value* <0.001), then between Day 3 and Day 4 (-*p-value* <0.001); the decrease from Day 4 until D15 was significant but slow (-*p-value* <0.05). It almost became undetectable after Day 4.

 The mean amount of Fluoride has also significantly decreased over time in the Equia Forte group (-*p-value* <0.001); it was 37,739 ± 1,377 ppm on D1 and 9,703 ± 2,757 ppm on D15; The decrease was progressive over time (-*p-value* <0.001).

**Figure 6:**
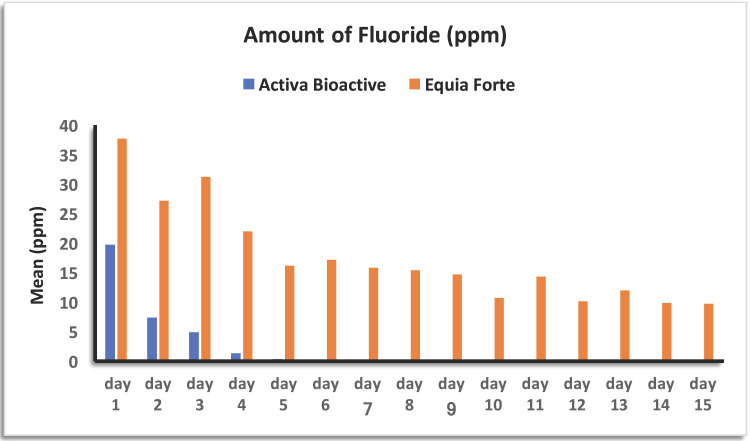
Amount of Fluoride released among groups within time.

However, the amount of Fluoride released was significantly greater in the Equia Forte group compared to the Activa Bioactive group on all days D1until D15 (-*p-value* <0.05) (Table 3, Fig. 6).

**Table 3:**
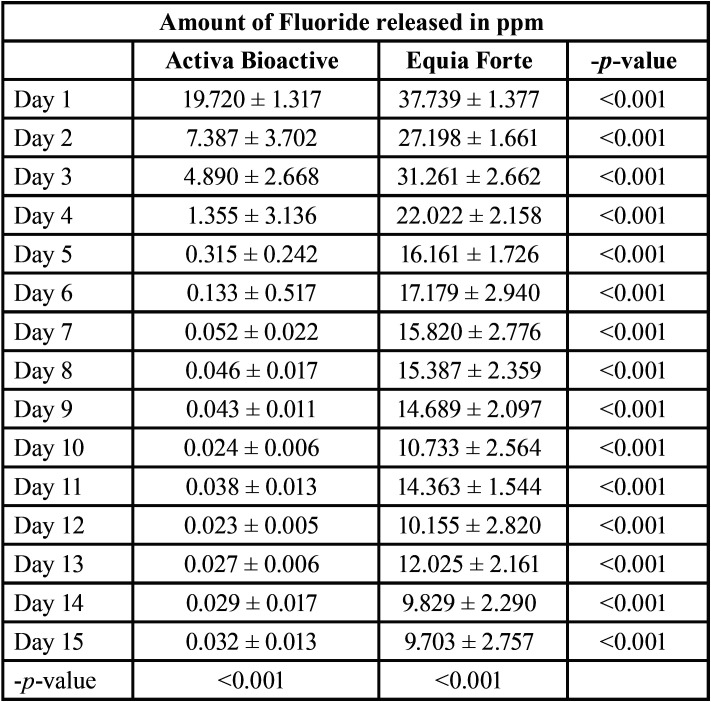
Mean amount of Fluoride among groups and within time.

## Discussion

-Shear Bond Strength 

Strong durable bond between dental biomaterials and tooth substrate are essential (2). Different mechanical tests have been proposed to assess the bonding performance of restorative materials. Shear bond strength test is a simple evaluation procedure used to test the bonding ability of adhesive materials to dental structure (30).

The results of the present study showed that Activa Bioactive restorative (AB) with adhesive exhibited similar shear bond strength in comparison to Filtek Z350 XT/Z350. This could be attributed to the ionic resin component which contains phosphate acid groups with antimicrobial properties that improve the interaction between the resin and the reactive glass fillers and enhance the interaction with tooth structure (31). In addition, AB is a blend of UDMA and other methacrylates (24); UDMA has improved mechanical properties over Bis-GMA and it improves shear force (32).

The results of our study are in discordance with a study by Sahar Abd El Halim (2018) in which a nanocomposite (Filtek™ Z350 XT) exhibits a higher shear bond strength than Activa BioActive Restorative with adhesive (33). It may be due to the adhesive used in the latter study (Adper Single Bond 2TM 3M/ESPE, Germany) which is not specific to Activa BioActive Restorative. In addition, a study by Latta, Mark A. *et al*. (2020) showed that AB presented lower SBS on dentin than other self-adhesive materials(Fuji II LC, Equia Forte et ASAR-MP4), these latters generated values that were lower than that generated with composite resin and a universal adhesive (34).

In this study, when Activa Bioactive Restorative was placed directly without a bonding agent, restorations were lost during fabrication of specimens. These findings contradict the self-adhesion capability claimed by the manufacturer. A study by Benetti AR, *et al*. showed as well that the self-adhesive property of ACTIVA Bioactive Restorative is nonexistent (35). In addition, The use of the AB restorative in Class II cavities (van Dijken JWV *et al*. 2019), applied as instructed by the manufacturer after a phosphoric acid pretreatment but without adhesive system, resulted in a non-acceptable very high failure frequency followed by postoperative sensitivity and secondary caries indicating the insufficient adhesion of AB to the tooth (25). Similarly, Philippe François *et al*. showed that Activa BioActive Restorative with a bonding agent presented higher SBS than Activa BioActive Restorative without a bonding agent (36).

-Fluoride release

The structure of a bioglass restorative may determine its bioactivity, as its inner porosity facilitates water flow through the material and the dissolution of the bioglass (37).

The breakup of the Si-O-Si bonds in the silicate network in an aqueous environment facilitates the mechanism of bioglass dissolution, which permits the rapid release of fluoride, calcium and silicon (38). Gandolfi *et al*. support the idea that the presence of hydrophilic resins, such as HEMA or triethylene glycol dimethacrylate (TEGDMA), could lead to hydrolytic disintegration of bioglass particles (37).

Measurements of fluoride release was studied every 24 h throughout 15 days. After a peak of fluoride release during the first 24 h, fluoride release from Equia forte decreased progressively over time ; this finding is comparable to that found by Maja Lezaja (2018) (39).

In addition, Activa Bioactive Restorative released most of their fluoride content during the first 4 days with a burst effect occurring in the first 24 h; this result is in accordance with that found by Alicja Porenczuk *et al*. (29).

Equia Forte group had higher fluoride release than Activa Bioactive group. Similar conclusions were provided by Mélissa Tiskaya *et al*. (40) who stated that AB released very few fluoride ions under different pH conditions and that there was no evidence of any apatite formation with Activa Bioactive. Hokii.Y *et al*. (41) who also compared the fluoride ion release from six restorative materials (GC Fuji AUtomix LC, Equia Forte Fil, Equia Forte Coat, Activa Bioactive Restorative, Beautiful II LS and GC Fuji Triage) under neutral and acidic pH conditions found that GC Fuji Automix LC, Equia Forte Fil, and GC Fuji had significant higher fluoride release properties compared to the other 3 products which include AB. 

Furthermore, Garoushi *et al*. found that AB released significantly less fluoride than another RMCIC (Fuji II lc) (42); this latter showed less fluoride release than Equia forte(39).

However, our finding is in discordance with that of May & Donly (2017) that showed that AB releases fluoride, does uptake it then re-releases it which could offer inhibition to caries at restoration margins (43).

Deionized water was used as the immersion solution as it is most frequently used in other studies and has been shown to facilitate more fluoride release than artificial saliva (44).

In clinical practice, it is advised to cover the surface of Equia restorations with Equia Forte coat that would probably act as a semi-permeable membrane, allowing partial fluoride release into the oral environment. However, low wear resistance of unfilled or very low filled resin liquid indicates that the coat layer would be worn during function leaving Equia exposed for unrestricted fluoride release. As the longevity of the protective coat in clinical conditions is unpredictable and individual, the present study design without any protective layer allowed measuring maximum fluoride release for the given sample size and shape (39).

In light of our findings, the three hypotheses were rejected.

## Conclusions

It may be concluded that, under the limitations of this study, the self-adhesive property of ACTIVA Bioactive Restorative is nonexistent.

Activa Bioactive Restorative with adhesive and Filtek Z350 XT/Z350 showed similar shear bond strength. The fluoride ion release profile of Activa BioActive Restorative was lower than that of the GIC Equia forte.

It would be interesting to undertake other comparative studies examining the recharge of fluorine ions after immersion of these materials, and at different pHs in solutions containing toothpastes or remineralizing mouthwashes.
